# Dynamic Research Support for Academic Libraries

**DOI:** 10.5195/jmla.2017.117

**Published:** 2017-01

**Authors:** Martha F. Earl

Libraries have long stood on the shifting sands of change. The key to survival has been to understand that change and meet it at the point of need. Starr Hoffman’s work provides a road map to that change as academic reference librarians make the shift to academic research support librarians. She emphasizes that the traditional role focused on discovery and instructional services has moved into the realm of a broader encompassment of exploration, learning, and collaboration. This evolving world of interdisciplinary cooperation across departments and disciplines in academia places librarians to use our essential expertise in collection development, metadata, reference, and instruction to meet the needs of our subject-based faculty colleagues. No matter what the library size or budget, librarians can contribute. Hoffman’s book is a valuable guide.

Although there exist many books on reference, research support and learning, and related initiatives—such as data services, digital humanities support, and data management—Hoffman seeks to provide illustrative examples of those services as correlated, emerging models of research support in one volume. Hoffman, head of planning and assessment at the University of Nevada, Las Vegas, knows her environment. This book does not limit itself to biomedical libraries or disciplines but spans the social sciences and humanities as well. Research is not the purview of one discipline or one country. Chapter contributors are international and come from both large academic institutions and smaller specialized projects. Research support is not “one size fits all.”

First, Hoffman defines the landscape and the essential terminology. In the introduction, she demonstrates a clear understanding of the nature of current reference and information services. Librarians find that reference sessions can quickly evolve into custom consultations on information literacy, the interpretation of quantitative statistics, methods of sharing research, or relative measurements of altmetrics. Librarians are also expanding liaison services to support open access publishing, data management, and digitalization projects. Instructional techniques are also shifting. Guided inquiry encourages students to ask questions to solve problems. Inquiry as conversation ties to the critical librarianship (critlib) movement in information literacy, encouraging students to think critically and challenge traditional authoritative sources.

Hoffman discusses the need for an exploratory culture, in which librarians are trained and encouraged to take creative risks in the redesign of services. A culture of assessment where all staff understand and contribute as partners creates an environment of proactive change; just as central are collaboration and engagement with the university, expanding the roles of liaison librarians to include academic support staff and student affairs, as well as offices of sponsored research, institutional repositories, and data management. Taking some of these services to the recommended level will require a reenvisioning by leadership. Hoffman notes that librarians shine when they focus on what institutions need most and what librarians do best. As in any other aspect of life, relationships are central to success.

The book is divided into three parts. Each part begins with an introduction discussing the theme of that section, followed by the case study chapters. Part 1, “Training and Infrastructure,” focuses on the role of library staff development and library physical spaces in research support, with chapters on library renovation to support digital scholarship, research into illustrated books in art history, and a digital scholarship pilot training project for librarians. Part 2, “Data Services and Data Literacy,” includes case studies on training researchers to manage data, creating the Digital Social Science Center at Columbia, and supporting geographic information systems (GIS) across nontraditional disciplines. Part 3, “Research as a Conversation,” discusses academic library initiatives to support the dissemination, discovery, and critical analysis of research, with chapters on implementing open access across a large university, an information literacy massive open online course (MOOC), and metadata enhancement through name authority for a university’s digital repository. Each chapter author clearly lays out practical information such as planning, staff needs, budgeting, time factors, processes, development, implementation, and assessment. A particularly useful component is the discussion of lessons learned. Each segment is well researched, with relevant, current lists of references. The book concludes with an index containing a minimum of cross-references.

Hoffman advises readers to use case studies in the book as launching pads to develop services specific to the readers’ institutions. The exceptionally clear writing and organization of this work by both the editor and the chapter authors make that possibility a likelihood. One might expect each chapter to follow the formatting of a research article, as each author clearly has compiled analyzable and publishable data as a result of their featured projects. Instead, the authors follow more of a program format, explaining processes and outcomes. The effect is that of a useful how-to manual.

This book is strongly recommended for academic librarians of any type seeking to update research services and support scholarly communications. For health sciences libraries, this book will prove most valuable in exploring roles and action plans to meet the growing needs of academic health sciences faculty and students. This book is also highly recommended for faculty and students in graduate schools of library and information sciences.

